# Tetra­kis(μ-6-hydr­oxy-1-naphthoato)bis­[(6-hydr­oxy-1-naphthoato)(1,10-phenanthroline)europium(III)] dihydrate

**DOI:** 10.1107/S1600536809046091

**Published:** 2009-11-14

**Authors:** Chun-Sen Liu, Min Hu, Qiang Zhang

**Affiliations:** a Zhengzhou University of Light Industry, Henan Provincial Key Laboratory of Surface & Interface Science, Henan, Zhengzhou 450002, People’s Republic of China

## Abstract

The title complex, [Eu_2_(C_11_H_7_O_3_)_6_(C_12_H_8_N_2_)_2_]·2H_2_O, has a centrosymmetric binuclear cage structure in which the two Eu^III^ ions are both nine-coordinated and bridged by 6-hy­droxy-1-naphthoate (*L*) ligands, with an Eu⋯ Eu separation of 4.1594 (4) Å. The remaining coordination sites are occupied by two N atoms from one 1,10-phenanthroline (phen) and two O atoms from an *L* ligand. The six 6-hydr­oxy-1-naphthoate groups coordinate each Eu^III^ atom in three different ways, namely μ_2_-η^1^:η^1^-bridging, μ_1_-η^1^:η^1^-chelating, and μ_2_-η^1^:η^2^-chelating/bridging modes. Adjacent discrete dinuclear units are linked into a two-dimensional sheet parallel to (011) by inter­molecular O—H⋯O hydrogen-bonding inter­actions. The sheets are cross-linked by water mol­ecules, forming a three-dimensional network. In addition, π–π stacking inter­actions, with a centroid–centroid separation of 3.547 (2) Å are observed.

## Related literature

For general background to functional rare-earth coordination complexes, see: Bünzli (2006 ▶); Edelmann (2009 ▶); Fang *et al.* (2006 ▶); Li & Yan (2009 ▶); Xu *et al.* (2009 ▶). For related structures, see: Bettencourt-Dias (2005 ▶); Bettencourt-Dias & Viswanathan (2006 ▶); Qu *et al.* (2005 ▶); Serre & Férey (2002 ▶); Wan *et al.* (2002 ▶); Yang *et al.* (2006 ▶); Ye *et al.* (2005 ▶); Zheng *et al.* (2005 ▶).
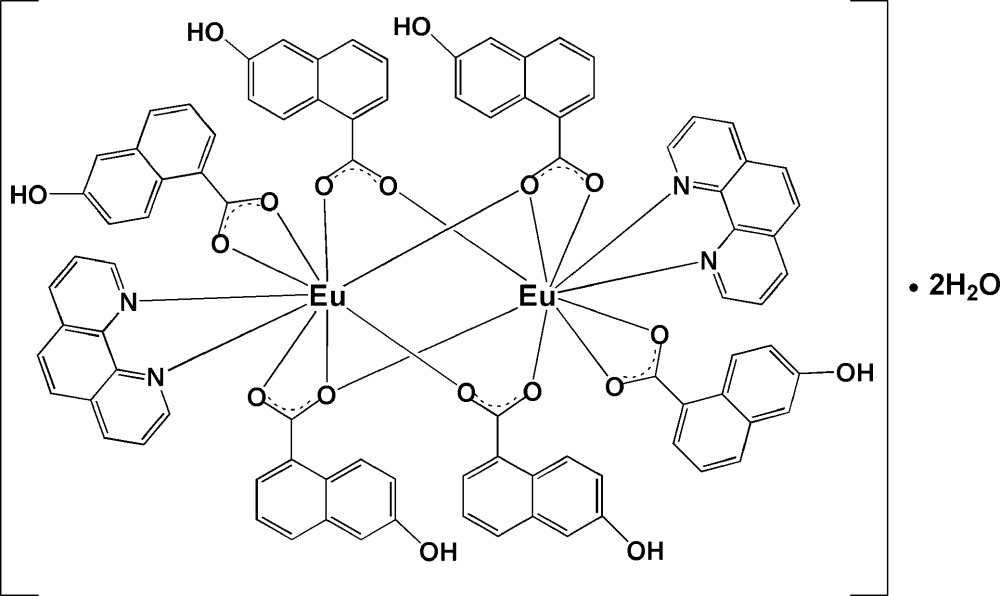


## Experimental

### 

#### Crystal data

[Eu_2_(C_11_H_7_O_3_)_6_(C_12_H_8_N_2_)_2_]·2H_2_O
*M* *_r_* = 1823.36
Triclinic, 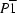

*a* = 11.7132 (9) Å
*b* = 12.7143 (10) Å
*c* = 14.8822 (12) Å
α = 65.790 (1)°
β = 88.276 (1)°
γ = 70.437 (1)°
*V* = 1889.8 (3) Å^3^
*Z* = 1
Mo *K*α radiation
μ = 1.73 mm^−1^
*T* = 296 K
0.30 × 0.21 × 0.17 mm

#### Data collection

Bruker SMART CCD area-detector diffractometer
Absorption correction: multi-scan (*SADABS*; Sheldrick, 1996 ▶) *T*_min_ = 0.626, *T*_max_ = 0.758
13943 measured reflections
6615 independent reflections
5910 reflections with *I* > 2σ(*I*)
*R*_int_ = 0.024

#### Refinement

*R*[*F* ^2^ > 2σ(*F* ^2^)] = 0.026
*wR*(*F* ^2^) = 0.057
*S* = 1.04
6615 reflections
526 parameters
H-atom parameters constrained
Δρ_max_ = 0.66 e Å^−3^
Δρ_min_ = −0.56 e Å^−3^

### 

Data collection: *SMART* (Bruker, 2007 ▶); cell refinement: *SAINT* (Bruker, 2007 ▶); data reduction: *SAINT*; program(s) used to solve structure: *SHELXS97* (Sheldrick, 2008 ▶); program(s) used to refine structure: *SHELXL97* (Sheldrick, 2008 ▶); molecular graphics: *SHELXTL* (Sheldrick, 2008 ▶); software used to prepare material for publication: *SHELXTL* and *PLATON* (Spek, 2009 ▶).

## Supplementary Material

Crystal structure: contains datablocks I, global. DOI: 10.1107/S1600536809046091/ci2947sup1.cif

Structure factors: contains datablocks I. DOI: 10.1107/S1600536809046091/ci2947Isup2.hkl

Additional supplementary materials:  crystallographic information; 3D view; checkCIF report

## Figures and Tables

**Table 1:** Selected bond lengths (Å)

Eu1—O5^i^	2.334 (2)
Eu1—O4	2.351 (2)
Eu1—O6	2.3680 (19)
Eu1—O2	2.411 (2)
Eu1—O1	2.464 (2)
Eu1—O3	2.471 (2)
Eu1—N1	2.591 (2)
Eu1—N2	2.593 (2)
Eu1—O4^i^	2.925 (2)

Symmetry code: (i) 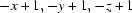.

**Table 2:** Hydrogen-bond geometry (Å, °)

*D*—H⋯*A*	*D*—H	H⋯*A*	*D*⋯*A*	*D*—H⋯*A*
O1*W*—H1*W*⋯O9^ii^	0.85	2.05	2.799 (4)	147
O1*W*—H2*W*⋯O8^iii^	0.85	2.28	3.050 (4)	150
O7—H7⋯O1*W*	0.82	1.89	2.670 (5)	159
O8—H8⋯O7^iv^	0.82	1.88	2.664 (5)	160
O9—H9⋯O3^v^	0.82	1.84	2.642 (3)	165

Symmetry codes: (ii) 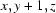; (iii) 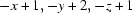; (iv) 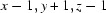; (v) 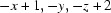.

**Fig. 1.**
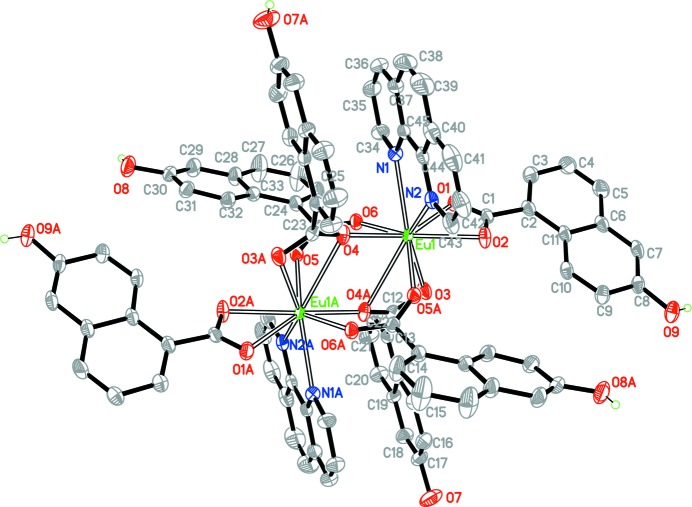
The molecular structure of the title complex. Displacement ellipsoids are drawn at the 30% probability level. Atom-labelled with the suffix A are generated by the symmetry operation (1 – x, 1 – y, 1 – z). Free water molecules have been omitted for clarity.

**Fig. 2.**
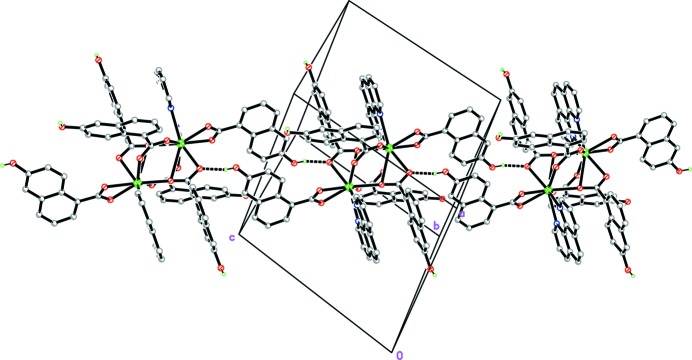
A view of a chain running along the [011], formed by O—H···O hydrogen-bonding interactions. For clarity, only H atoms involved in the interactions are shown.

**Fig. 3.**
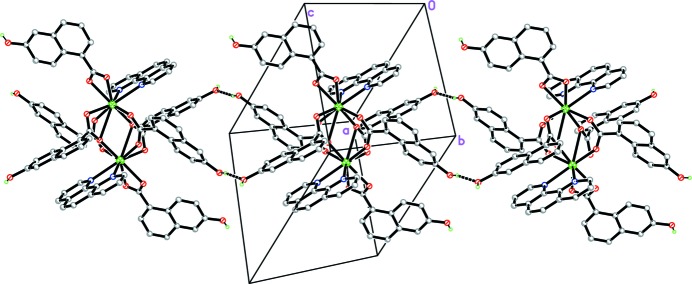
A view of a chain running along the [111], formed by O—H···O hydrogen-bonding interactions. For clarity, only H atoms involved in the interactions are shown.

**Fig. 4.**
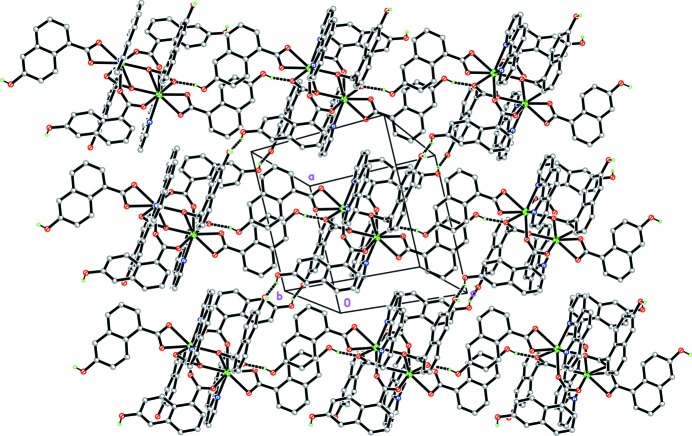
A view of the two-dimensional network of dinuclear complex, formed by O—H···O hydrogen-bonding interactions (solid dashed lines). For clarity, only H atoms involved in the interactions are shown.

## supplementary crystallographic information

### Comment

In recent years, the rational design and synthesis of functional rare-earth (RE) coordination complexes with various N– and/or O-donor ligands has attracted great interest because of not only their fascinating structural diversities but also their potential applications as functional materials, for example, optical materials, electronic materials, catalytic materials, and molecular-based magnets (Bünzli, 2006; Edelmann, 2009; Fang *et al.*, 2006; Li & Yan, 2009; Xu *et al.*, 2009). In this field, the choice of suitable organic ligands favoring structure-specific self-assembly is crucial for the construction of prospective coordination structures with relevant properties and functions. Among various ligands, the versatile carboxylic acid ligands, especially the benzene- and naphthalene-based di- and multi-carboxylic acids, have been well used in the preparation of various rare-earth (RE) complexes owing to their rich coordination modes (Bettencourt-Dias, 2005; Bettencourt-Dias & Viswanathan, 2006; Qu *et al.*, 2005; Serre & Férey, 2002; Yang *et al.*, 2006). However, far less common has been the use of naphthalene-based monocarboxylic acids, such as the acid used herein, 6-hydroxy-1-naphthoic acid (H*L*). Besides, the introduction of 2,2'-bipyridyl-like bidentate chelating molecules (2,2'-bipyridine or 1,10-phenanthroline) into the reaction systems including various carboxylic acid ligands, as auxiliary ligands, can generate some interesting coordination architectures (Ye *et al.*, 2005). We report here the crystal structure of the title complex (I), a Eu^III ^complex with mixed 6-hydroxy-1-naphthoic acid (*L*) and chelating 1,10-phenanthroline (phen) ligands.

The structure of complex (I) consists of a centrosymmetric dinuclear unit [Eu_2_(*L*)_6_(phen)_2_] and two free water molecules. The Eu^III^ ion is nine-coordinated by two N-atom donors from one chelating phen ligand and seven O atoms from five distinct *L *ligands (Fig. 1). The Eu—O distances are in the range of 2.334 (2)–2.925 (2) Å, which are normal and in agreement with those found in other carboxylato-containing Eu^III^ complexes (Zheng *et al.*, 2005; Wan *et al.*, 2002). The phen ligand acts as a typical chelating ligand coordinating to the Eu^III^ ion with Eu—N bond distances of 2.591 (2) and 2.593 (2) Å, and an N—Eu—N angle of 63.57 (8)°. For *L*, there exists three different kinds of carboxylate coordination modes with the Eu^III^ center, namely *syn-syn* bridging (µ_2_-η^1^:η^1^-bridging), symmetric bidentate chelate (µ_1_-η^1^:η^1^-chelating), and tridentate chelating/bridging (µ_2_-η^1^:η^2^-chelating/bridging). In this case two Eu^III^ ions are connected to form an eight-membered ring [Eu1/O6/C23/O5/Eu1A/O6A/C23A/O5A], as well as a four-membered ring [Eu1/O4/Eu1A/O4A]. The non-bonding Eu1···Eu1A separation is 4.1594 (4) Å (symmetry operation A = 1 - *x*, 1 - *y*, 1 - *z*).

Adjacent dinuclear [Eu_2_(*L*)_6_(phen)_2_] units are linked into two different chains running along the [011] (Fig.2) and [111] (Fig. 3) and thus generating a two-dimensional sheet parallel to the (011) by intermolecular O—H···O hydrogen-bonding interactions (Table 1) between different *L *ligands (Fig. 4). The sheets are cross-linked via O—H···O hydrogen bonds involving the water molecules. In addition, intermolecular π–π stacking interactions are observed between the N2/C43/C42/C41/C40/C44 pyridine rings of the phen ligands at (x, y, z) and (1-x, -y, 1-z), with a centroid-to-centroid separation of 3.547 (2) Å.

### Experimental

A mixed solution of 6-hydroxy-1-naphthoic acid (0.05 mmol) and 1,10-phenanthroline (0.05 mmol) in CH_3_OH (10 ml) in the presence of excess 2,6-dimethylpyridine (*ca* 0.05 ml for adjusting the pH value to basic condition) was carefully layered on top of a H_2_O solution (15 ml) of Eu(ClO_4_)_3_ (0.1 mmol) in a test tube. Yellow single crystals suitable for X-ray analysis of the title complex (I) appeared at the tube wall after *ca* two weeks at room temperature. Yield: ~40% based on 6-hydroxy-1-naphthoic acid. Elemental analysis calculated for C_45_H_31_EuN_2_O_10_: C 59.28, H 3.43, N 3.07%; found: C 59.36, H 3.50, N 3.03%.

### Refinement

The water H atoms were located in a difference Fourier map and treated as riding on their parent atoms, with O-H = 0.85 Å and *U*_iso_(H) = 1.2*U*_eq_(O). C-bound H-atoms were included in calculated positions and treated as riding atoms: C-H = 0.93 Å with *U*_iso_(H) = 1.2 *U*_eq_(C).

### Crystal data

**Table:**

[Eu_2_(C_11_H_7_O_3_)_6_(C_12_H_8_N_2_)_2_]·2H_2_O	*Z* = 1
*M**_r_* = 1823.36	*F*(000) = 916
Triclinic, *P*1	*D*_x_ = 1.602 Mg m^−^^3^
Hall symbol: -P 1	Mo *K*α radiation, λ = 0.71073 Å
*a* = 11.7132 (9) Å	Cell parameters from 6015 reflections
*b* = 12.7143 (10) Å	θ = 2.5–25.6°
*c* = 14.8822 (12) Å	µ = 1.73 mm^−^^1^
α = 65.790 (1)°	*T* = 296 K
β = 88.276 (1)°	Block, yellow
γ = 70.437 (1)°	0.30 × 0.21 × 0.17 mm
*V* = 1889.8 (3) Å^3^	

### Data collection

**Table:**

Bruker SMART CCD area-detector diffractometer	6615 independent reflections
Radiation source: fine-focus sealed tube	5910 reflections with *I* > 2σ(*I*)
graphite	*R*_int_ = 0.024
φ and ω scans	θ_max_ = 25.0°, θ_min_ = 2.3°
Absorption correction: multi-scan (*SADABS*; Sheldrick, 1996)	*h* = −13→13
*T*_min_ = 0.626, *T*_max_ = 0.758	*k* = −15→14
13943 measured reflections	*l* = −17→17

### Refinement

**Table:**

Refinement on *F*^2^	Primary atom site location: structure-invariant direct methods
Least-squares matrix: full	Secondary atom site location: difference Fourier map
*R*[*F*^2^ > 2σ(*F*^2^)] = 0.026	Hydrogen site location: inferred from neighbouring sites
*wR*(*F*^2^) = 0.057	H-atom parameters constrained
*S* = 1.04	*w* = 1/[σ^2^(*F*_o_^2^) + (0.0253*P*)^2^ + 0.306*P*] where *P* = (*F*_o_^2^ + 2*F*_c_^2^)/3
6615 reflections	(Δ/σ)_max_ = 0.002
526 parameters	Δρ_max_ = 0.66 e Å^−^^3^
0 restraints	Δρ_min_ = −0.56 e Å^−^^3^

### Special details

**Table:**

Geometry. All e.s.d.'s (except the e.s.d. in the dihedral angle between two l.s. planes) are estimated using the full covariance matrix. The cell e.s.d.'s are taken into account individually in the estimation of e.s.d.'s in distances, angles and torsion angles; correlations between e.s.d.'s in cell parameters are only used when they are defined by crystal symmetry. An approximate (isotropic) treatment of cell e.s.d.'s is used for estimating e.s.d.'s involving l.s. planes.
Refinement. Refinement of *F*^2^ against ALL reflections. The weighted *R*-factor *wR* and goodness of fit *S* are based on *F*^2^, conventional *R*-factors *R* are based on *F*, with *F* set to zero for negative *F*^2^. The threshold expression of *F*^2^ > σ(*F*^2^) is used only for calculating *R*-factors(gt) *etc*. and is not relevant to the choice of reflections for refinement. *R*-factors based on *F*^2^ are statistically about twice as large as those based on *F*, and *R*- factors based on ALL data will be even larger.

### Fractional atomic coordinates and isotropic or equivalent isotropic displacement parameters (Å^2^)

**Table:**

	*x*	*y*	*z*	*U*_iso_*/*U*_eq_	
Eu1	0.415191 (13)	0.370163 (13)	0.555623 (10)	0.02734 (6)	
C1	0.3329 (3)	0.2031 (3)	0.7084 (2)	0.0355 (7)	
C2	0.2925 (3)	0.1144 (3)	0.7940 (2)	0.0411 (8)	
C3	0.1694 (3)	0.1376 (4)	0.7937 (3)	0.0572 (10)	
H3A	0.1152	0.2070	0.7424	0.069*	
C4	0.1236 (4)	0.0577 (4)	0.8698 (3)	0.0668 (12)	
H4A	0.0401	0.0730	0.8671	0.080*	
C5	0.2004 (4)	−0.0405 (4)	0.9465 (3)	0.0622 (11)	
H5A	0.1682	−0.0909	0.9968	0.075*	
C6	0.3281 (3)	−0.0695 (3)	0.9532 (2)	0.0439 (8)	
C7	0.4075 (3)	−0.1704 (3)	1.0349 (2)	0.0472 (9)	
H7A	0.3757	−0.2195	1.0865	0.057*	
C8	0.5305 (3)	−0.1969 (3)	1.0391 (2)	0.0420 (8)	
C9	0.5790 (3)	−0.1237 (3)	0.9611 (2)	0.0472 (8)	
H9A	0.6631	−0.1435	0.9636	0.057*	
C10	0.5050 (3)	−0.0246 (3)	0.8822 (2)	0.0412 (8)	
H10A	0.5396	0.0233	0.8322	0.049*	
C11	0.3765 (3)	0.0082 (3)	0.8737 (2)	0.0390 (7)	
C12	0.4939 (3)	0.4799 (3)	0.6816 (2)	0.0386 (7)	
C13	0.4849 (3)	0.5442 (3)	0.7483 (2)	0.0381 (7)	
C14	0.5856 (3)	0.5138 (3)	0.8151 (2)	0.0364 (7)	
C15	0.7022 (3)	0.4254 (3)	0.8225 (2)	0.0456 (8)	
H15A	0.7149	0.3856	0.7808	0.055*	
C16	0.7955 (3)	0.3983 (3)	0.8900 (3)	0.0537 (9)	
H16A	0.8712	0.3399	0.8944	0.064*	
C17	0.7784 (3)	0.4578 (3)	0.9528 (3)	0.0560 (10)	
C18	0.6692 (3)	0.5442 (3)	0.9478 (3)	0.0540 (9)	
H18A	0.6595	0.5836	0.9896	0.065*	
C19	0.5706 (3)	0.5740 (3)	0.8790 (2)	0.0425 (8)	
C20	0.4563 (3)	0.6639 (3)	0.8721 (3)	0.0534 (9)	
H20A	0.4461	0.7046	0.9130	0.064*	
C21	0.3608 (3)	0.6917 (4)	0.8058 (3)	0.0613 (10)	
H21A	0.2861	0.7514	0.8015	0.074*	
C22	0.3753 (3)	0.6300 (3)	0.7442 (3)	0.0534 (9)	
H22A	0.3095	0.6480	0.7002	0.064*	
C23	0.2781 (3)	0.6807 (3)	0.4551 (2)	0.0356 (7)	
C24	0.1583 (3)	0.7857 (3)	0.4238 (2)	0.0413 (8)	
C25	0.0743 (3)	0.7802 (4)	0.4887 (3)	0.0709 (13)	
H25A	0.0888	0.7086	0.5468	0.085*	
C26	−0.0343 (4)	0.8810 (4)	0.4695 (4)	0.108 (2)	
H26A	−0.0907	0.8761	0.5150	0.130*	
C27	−0.0572 (4)	0.9860 (4)	0.3841 (4)	0.0933 (17)	
H27A	−0.1288	1.0526	0.3725	0.112*	
C28	0.0259 (3)	0.9956 (3)	0.3128 (3)	0.0559 (10)	
C29	0.0020 (3)	1.1045 (3)	0.2234 (3)	0.0591 (10)	
H29A	−0.0678	1.1728	0.2124	0.071*	
C30	0.0798 (3)	1.1095 (3)	0.1540 (3)	0.0504 (9)	
C31	0.1842 (3)	1.0078 (3)	0.1691 (2)	0.0487 (9)	
H31A	0.2354	1.0112	0.1198	0.058*	
C32	0.2123 (3)	0.9030 (3)	0.2558 (2)	0.0439 (8)	
H32A	0.2835	0.8368	0.2651	0.053*	
C33	0.1358 (3)	0.8934 (3)	0.3311 (2)	0.0399 (8)	
C34	0.1302 (3)	0.4750 (3)	0.4221 (3)	0.0511 (9)	
H34A	0.1139	0.5224	0.4579	0.061*	
C35	0.0351 (3)	0.4935 (4)	0.3564 (3)	0.0626 (11)	
H35A	−0.0424	0.5508	0.3492	0.075*	
C36	0.0593 (4)	0.4255 (4)	0.3035 (3)	0.0680 (12)	
H36A	−0.0022	0.4368	0.2586	0.082*	
C37	0.1750 (4)	0.3386 (4)	0.3153 (3)	0.0560 (10)	
C38	0.2077 (5)	0.2633 (5)	0.2623 (3)	0.0789 (14)	
H38A	0.1489	0.2719	0.2166	0.095*	
C39	0.3197 (5)	0.1811 (5)	0.2764 (3)	0.0818 (14)	
H39A	0.3377	0.1344	0.2399	0.098*	
C40	0.4123 (4)	0.1636 (3)	0.3465 (3)	0.0573 (10)	
C41	0.5305 (4)	0.0754 (4)	0.3656 (3)	0.0681 (12)	
H41A	0.5522	0.0269	0.3307	0.082*	
C42	0.6124 (4)	0.0618 (3)	0.4354 (3)	0.0650 (12)	
H42A	0.6909	0.0039	0.4486	0.078*	
C43	0.5789 (3)	0.1348 (3)	0.4875 (3)	0.0520 (9)	
H43A	0.6360	0.1227	0.5363	0.062*	
C44	0.3857 (3)	0.2351 (3)	0.4011 (2)	0.0389 (8)	
C45	0.2655 (3)	0.3247 (3)	0.3845 (2)	0.0390 (8)	
N1	0.2417 (2)	0.3944 (2)	0.43657 (18)	0.0380 (6)	
N2	0.4692 (2)	0.2208 (2)	0.47064 (19)	0.0378 (6)	
O1	0.25998 (19)	0.3115 (2)	0.66020 (16)	0.0501 (6)	
O2	0.4394 (2)	0.16976 (18)	0.68524 (16)	0.0471 (6)	
O3	0.4570 (2)	0.3902 (2)	0.70848 (15)	0.0518 (6)	
O4	0.4683 (2)	0.48225 (19)	0.40107 (15)	0.0448 (5)	
O5	0.37166 (18)	0.70586 (18)	0.42525 (16)	0.0418 (5)	
O6	0.27937 (18)	0.57429 (18)	0.51237 (15)	0.0395 (5)	
O7	0.8780 (3)	0.4249 (3)	1.0199 (2)	0.0842 (10)	
H7	0.8599	0.4675	1.0509	0.126*	
O8	0.0629 (2)	1.2126 (2)	0.06669 (19)	0.0710 (8)	
H8	0.0006	1.2682	0.0649	0.106*	
O9	0.6148 (2)	−0.2937 (2)	1.11577 (16)	0.0572 (7)	
H9	0.5805	−0.3185	1.1648	0.086*	
O1W	0.8555 (2)	0.5914 (3)	1.0912 (2)	0.0806 (9)	
H1W	0.7933	0.6013	1.1220	0.097*	
H2W	0.8754	0.6550	1.0650	0.097*	

### Atomic displacement parameters (Å^2^)

**Table:**

	*U*^11^	*U*^22^	*U*^33^	*U*^12^	*U*^13^	*U*^23^
Eu1	0.02963 (9)	0.02244 (8)	0.02384 (8)	−0.00843 (6)	0.00206 (6)	−0.00460 (6)
C1	0.0463 (19)	0.0347 (18)	0.0319 (17)	−0.0226 (16)	0.0079 (15)	−0.0139 (15)
C2	0.052 (2)	0.0419 (19)	0.0371 (18)	−0.0274 (17)	0.0127 (16)	−0.0157 (16)
C3	0.056 (2)	0.062 (2)	0.046 (2)	−0.033 (2)	0.0063 (18)	−0.0076 (18)
C4	0.055 (2)	0.080 (3)	0.058 (3)	−0.042 (2)	0.011 (2)	−0.008 (2)
C5	0.070 (3)	0.075 (3)	0.048 (2)	−0.050 (2)	0.020 (2)	−0.014 (2)
C6	0.063 (2)	0.043 (2)	0.0358 (18)	−0.0323 (18)	0.0151 (17)	−0.0160 (16)
C7	0.076 (3)	0.042 (2)	0.0326 (18)	−0.0370 (19)	0.0170 (18)	−0.0117 (16)
C8	0.064 (2)	0.0336 (18)	0.0280 (17)	−0.0199 (17)	0.0107 (16)	−0.0112 (15)
C9	0.055 (2)	0.045 (2)	0.0357 (19)	−0.0189 (18)	0.0137 (16)	−0.0118 (16)
C10	0.057 (2)	0.0357 (18)	0.0314 (17)	−0.0225 (16)	0.0160 (16)	−0.0109 (15)
C11	0.058 (2)	0.0370 (18)	0.0314 (17)	−0.0280 (16)	0.0149 (15)	−0.0149 (15)
C12	0.0442 (19)	0.0394 (19)	0.0265 (16)	−0.0155 (16)	0.0006 (14)	−0.0079 (14)
C13	0.0463 (19)	0.0374 (18)	0.0307 (17)	−0.0208 (16)	0.0065 (14)	−0.0101 (14)
C14	0.0437 (19)	0.0288 (17)	0.0323 (17)	−0.0149 (15)	0.0043 (14)	−0.0073 (14)
C15	0.050 (2)	0.0366 (19)	0.046 (2)	−0.0151 (16)	0.0074 (17)	−0.0129 (16)
C16	0.046 (2)	0.042 (2)	0.061 (2)	−0.0050 (17)	−0.0003 (18)	−0.0172 (19)
C17	0.055 (2)	0.043 (2)	0.056 (2)	−0.0090 (19)	−0.0157 (19)	−0.0140 (19)
C18	0.055 (2)	0.051 (2)	0.055 (2)	−0.0113 (19)	−0.0063 (18)	−0.0257 (19)
C19	0.045 (2)	0.0405 (19)	0.0389 (18)	−0.0144 (16)	−0.0036 (15)	−0.0145 (16)
C20	0.054 (2)	0.057 (2)	0.055 (2)	−0.0115 (19)	0.0031 (18)	−0.034 (2)
C21	0.041 (2)	0.068 (3)	0.068 (3)	−0.0015 (19)	0.0005 (19)	−0.036 (2)
C22	0.044 (2)	0.063 (2)	0.053 (2)	−0.0135 (19)	−0.0047 (17)	−0.029 (2)
C23	0.0345 (17)	0.0280 (17)	0.0356 (17)	−0.0064 (14)	0.0008 (14)	−0.0087 (14)
C24	0.0289 (16)	0.0310 (17)	0.052 (2)	−0.0073 (14)	0.0054 (15)	−0.0094 (15)
C25	0.047 (2)	0.048 (2)	0.072 (3)	−0.0024 (19)	0.018 (2)	0.006 (2)
C26	0.063 (3)	0.073 (3)	0.107 (4)	0.010 (3)	0.047 (3)	0.009 (3)
C27	0.054 (3)	0.055 (3)	0.105 (4)	0.012 (2)	0.033 (3)	0.001 (3)
C28	0.0325 (19)	0.041 (2)	0.065 (2)	−0.0042 (16)	0.0066 (17)	−0.0018 (18)
C29	0.037 (2)	0.037 (2)	0.070 (3)	0.0006 (16)	−0.0014 (19)	−0.0016 (19)
C30	0.043 (2)	0.042 (2)	0.047 (2)	−0.0131 (17)	−0.0076 (17)	−0.0019 (17)
C31	0.0405 (19)	0.050 (2)	0.042 (2)	−0.0127 (17)	0.0030 (16)	−0.0095 (17)
C32	0.0358 (18)	0.0393 (19)	0.046 (2)	−0.0074 (15)	0.0023 (16)	−0.0129 (16)
C33	0.0280 (16)	0.0324 (18)	0.050 (2)	−0.0062 (14)	0.0017 (15)	−0.0116 (15)
C34	0.0355 (19)	0.052 (2)	0.059 (2)	−0.0102 (17)	−0.0020 (17)	−0.0201 (19)
C35	0.040 (2)	0.064 (3)	0.066 (3)	−0.0164 (19)	−0.0068 (19)	−0.011 (2)
C36	0.070 (3)	0.074 (3)	0.051 (2)	−0.039 (3)	−0.018 (2)	−0.005 (2)
C37	0.076 (3)	0.059 (2)	0.038 (2)	−0.042 (2)	−0.0043 (19)	−0.0102 (18)
C38	0.108 (4)	0.101 (4)	0.058 (3)	−0.060 (3)	0.003 (3)	−0.043 (3)
C39	0.131 (4)	0.090 (4)	0.068 (3)	−0.063 (4)	0.024 (3)	−0.056 (3)
C40	0.088 (3)	0.049 (2)	0.053 (2)	−0.039 (2)	0.031 (2)	−0.0292 (19)
C41	0.100 (4)	0.052 (3)	0.078 (3)	−0.039 (3)	0.049 (3)	−0.045 (2)
C42	0.065 (3)	0.039 (2)	0.094 (3)	−0.018 (2)	0.040 (2)	−0.034 (2)
C43	0.051 (2)	0.0339 (19)	0.071 (3)	−0.0171 (17)	0.0185 (19)	−0.0210 (18)
C44	0.059 (2)	0.0321 (18)	0.0330 (17)	−0.0274 (17)	0.0149 (16)	−0.0129 (14)
C45	0.053 (2)	0.0360 (18)	0.0305 (17)	−0.0260 (16)	0.0038 (15)	−0.0087 (14)
N1	0.0382 (15)	0.0372 (15)	0.0362 (15)	−0.0144 (13)	0.0014 (12)	−0.0124 (12)
N2	0.0421 (15)	0.0268 (14)	0.0415 (15)	−0.0131 (12)	0.0091 (13)	−0.0112 (12)
O1	0.0372 (13)	0.0429 (14)	0.0467 (14)	−0.0121 (11)	0.0042 (11)	0.0014 (11)
O2	0.0591 (15)	0.0282 (12)	0.0416 (13)	−0.0132 (11)	0.0227 (12)	−0.0062 (10)
O3	0.0864 (18)	0.0552 (15)	0.0287 (12)	−0.0477 (14)	0.0125 (12)	−0.0144 (11)
O4	0.0575 (14)	0.0386 (13)	0.0289 (12)	−0.0168 (11)	0.0123 (11)	−0.0062 (10)
O5	0.0313 (12)	0.0288 (12)	0.0507 (14)	−0.0079 (10)	0.0008 (10)	−0.0051 (10)
O6	0.0363 (12)	0.0281 (12)	0.0441 (13)	−0.0094 (10)	0.0057 (10)	−0.0076 (10)
O7	0.0682 (18)	0.066 (2)	0.096 (2)	0.0060 (15)	−0.0412 (17)	−0.0339 (17)
O8	0.0625 (18)	0.0504 (17)	0.0587 (17)	−0.0106 (14)	−0.0044 (14)	0.0091 (14)
O9	0.0728 (17)	0.0471 (15)	0.0338 (13)	−0.0192 (13)	0.0120 (12)	−0.0022 (12)
O1W	0.0645 (18)	0.088 (2)	0.083 (2)	−0.0247 (17)	−0.0049 (16)	−0.0317 (18)

### Geometric parameters (Å, °)

**Table:**

Eu1—O5^i^	2.334 (2)	C23—O5	1.260 (3)
Eu1—O4	2.351 (2)	C23—O6	1.265 (3)
Eu1—O6	2.3680 (19)	C23—C24	1.497 (4)
Eu1—O2	2.411 (2)	C24—C25	1.356 (5)
Eu1—O1	2.464 (2)	C24—C33	1.442 (4)
Eu1—O3	2.471 (2)	C25—C26	1.404 (5)
Eu1—N1	2.591 (2)	C25—H25A	0.93
Eu1—N2	2.593 (2)	C26—C27	1.363 (6)
Eu1—C1	2.809 (3)	C26—H26A	0.93
Eu1—O4^i^	2.925 (2)	C27—C28	1.414 (5)
Eu1—C12	3.070 (3)	C27—H27A	0.93
Eu1—Eu1^i^	4.1594 (4)	C28—C29	1.420 (5)
C1—O1	1.257 (4)	C28—C33	1.425 (4)
C1—O2	1.263 (4)	C29—C30	1.352 (5)
C1—C2	1.503 (4)	C29—H29A	0.93
C2—C3	1.372 (5)	C30—O8	1.375 (4)
C2—C11	1.437 (4)	C30—C31	1.392 (4)
C3—C4	1.409 (5)	C31—C32	1.367 (4)
C3—H3A	0.93	C31—H31A	0.93
C4—C5	1.346 (5)	C32—C33	1.406 (4)
C4—H4A	0.93	C32—H32A	0.93
C5—C6	1.412 (5)	C34—N1	1.320 (4)
C5—H5A	0.93	C34—C35	1.399 (5)
C6—C7	1.406 (5)	C34—H34A	0.93
C6—C11	1.439 (4)	C35—C36	1.352 (6)
C7—C8	1.362 (5)	C35—H35A	0.93
C7—H7A	0.93	C36—C37	1.393 (6)
C8—O9	1.376 (4)	C36—H36A	0.93
C8—C9	1.402 (4)	C37—C45	1.416 (4)
C9—C10	1.354 (4)	C37—C38	1.429 (6)
C9—H9A	0.93	C38—C39	1.333 (6)
C10—C11	1.415 (4)	C38—H38A	0.93
C10—H10A	0.93	C39—C40	1.424 (6)
C12—O4^i^	1.252 (3)	C39—H39A	0.93
C12—O3	1.261 (4)	C40—C41	1.406 (5)
C12—C13	1.506 (4)	C40—C44	1.410 (5)
C13—C22	1.361 (4)	C41—C42	1.353 (6)
C13—C14	1.406 (4)	C41—H41A	0.93
C14—C19	1.420 (4)	C42—C43	1.395 (5)
C14—C15	1.422 (4)	C42—H42A	0.93
C15—C16	1.361 (5)	C43—N2	1.327 (4)
C15—H15A	0.93	C43—H43A	0.93
C16—C17	1.398 (5)	C44—N2	1.359 (4)
C16—H16A	0.93	C44—C45	1.434 (4)
C17—C18	1.359 (5)	C45—N1	1.360 (4)
C17—O7	1.390 (4)	O4—C12^i^	1.252 (3)
C18—C19	1.409 (4)	O4—Eu1^i^	2.925 (2)
C18—H18A	0.93	O5—Eu1^i^	2.334 (2)
C19—C20	1.413 (4)	O7—H7	0.82
C20—C21	1.366 (5)	O8—H8	0.82
C20—H20A	0.93	O9—H9	0.82
C21—C22	1.405 (5)	O1W—H1W	0.85
C21—H21A	0.93	O1W—H2W	0.85
C22—H22A	0.93		
			
O5^i^—Eu1—O4	74.98 (7)	C19—C14—C15	117.9 (3)
O5^i^—Eu1—O6	129.05 (7)	C16—C15—C14	120.8 (3)
O4—Eu1—O6	77.08 (7)	C16—C15—H15A	119.6
O5^i^—Eu1—O2	84.06 (7)	C14—C15—H15A	119.6
O4—Eu1—O2	146.76 (8)	C15—C16—C17	120.4 (3)
O6—Eu1—O2	135.53 (7)	C15—C16—H16A	119.8
O5^i^—Eu1—O1	134.24 (7)	C17—C16—H16A	119.8
O4—Eu1—O1	150.25 (8)	C18—C17—O7	121.9 (3)
O6—Eu1—O1	84.24 (7)	C18—C17—C16	121.1 (3)
O2—Eu1—O1	53.17 (7)	O7—C17—C16	117.0 (3)
O5^i^—Eu1—O3	80.21 (8)	C17—C18—C19	119.9 (3)
O4—Eu1—O3	123.18 (7)	C17—C18—H18A	120.1
O6—Eu1—O3	80.71 (8)	C19—C18—H18A	120.1
O2—Eu1—O3	76.85 (7)	C18—C19—C20	120.8 (3)
O1—Eu1—O3	75.27 (8)	C18—C19—C14	120.0 (3)
O5^i^—Eu1—N1	136.29 (8)	C20—C19—C14	119.2 (3)
O4—Eu1—N1	79.35 (8)	C21—C20—C19	120.6 (3)
O6—Eu1—N1	76.81 (8)	C21—C20—H20A	119.7
O2—Eu1—N1	99.70 (8)	C19—C20—H20A	119.7
O1—Eu1—N1	73.93 (8)	C20—C21—C22	120.0 (3)
O3—Eu1—N1	143.30 (8)	C20—C21—H21A	120.0
O5^i^—Eu1—N2	76.11 (8)	C22—C21—H21A	120.0
O4—Eu1—N2	75.88 (7)	C13—C22—C21	120.6 (3)
O6—Eu1—N2	135.20 (8)	C13—C22—H22A	119.7
O2—Eu1—N2	74.18 (7)	C21—C22—H22A	119.7
O1—Eu1—N2	102.94 (8)	O5—C23—O6	124.5 (3)
O3—Eu1—N2	144.07 (8)	O5—C23—C24	117.3 (3)
N1—Eu1—N2	63.57 (8)	O6—C23—C24	118.1 (3)
O5^i^—Eu1—C1	109.21 (8)	C25—C24—C33	120.7 (3)
O4—Eu1—C1	163.08 (8)	C25—C24—C23	117.8 (3)
O6—Eu1—C1	109.84 (8)	C33—C24—C23	121.4 (3)
O2—Eu1—C1	26.63 (8)	C24—C25—C26	121.0 (3)
O1—Eu1—C1	26.56 (8)	C24—C25—H25A	119.5
O3—Eu1—C1	73.66 (8)	C26—C25—H25A	119.5
N1—Eu1—C1	87.13 (8)	C27—C26—C25	120.0 (4)
N2—Eu1—C1	89.05 (8)	C27—C26—H26A	120.0
O5^i^—Eu1—O4^i^	64.62 (7)	C25—C26—H26A	120.0
O4—Eu1—O4^i^	76.48 (7)	C26—C27—C28	121.3 (4)
O6—Eu1—O4^i^	67.92 (7)	C26—C27—H27A	119.3
O2—Eu1—O4^i^	117.37 (7)	C28—C27—H27A	119.3
O1—Eu1—O4^i^	117.53 (7)	C27—C28—C29	121.5 (3)
O3—Eu1—O4^i^	46.70 (6)	C27—C28—C33	119.0 (3)
N1—Eu1—O4^i^	140.72 (7)	C29—C28—C33	119.4 (3)
N2—Eu1—O4^i^	136.47 (7)	C30—C29—C28	120.5 (3)
C1—Eu1—O4^i^	120.32 (7)	C30—C29—H29A	119.8
O5^i^—Eu1—C12	74.44 (8)	C28—C29—H29A	119.8
O4—Eu1—C12	100.14 (8)	C29—C30—O8	123.3 (3)
O6—Eu1—C12	69.68 (8)	C29—C30—C31	120.5 (3)
O2—Eu1—C12	98.67 (8)	O8—C30—C31	116.2 (3)
O1—Eu1—C12	94.69 (8)	C32—C31—C30	120.6 (3)
O3—Eu1—C12	23.25 (7)	C32—C31—H31A	119.7
N1—Eu1—C12	145.58 (8)	C30—C31—H31A	119.7
N2—Eu1—C12	150.26 (8)	C31—C32—C33	121.3 (3)
C1—Eu1—C12	96.76 (8)	C31—C32—H32A	119.3
O4^i^—Eu1—C12	23.95 (7)	C33—C32—H32A	119.3
O5^i^—Eu1—Eu1^i^	63.39 (5)	C32—C33—C28	117.5 (3)
O4—Eu1—Eu1^i^	43.14 (5)	C32—C33—C24	124.6 (3)
O6—Eu1—Eu1^i^	67.00 (5)	C28—C33—C24	117.8 (3)
O2—Eu1—Eu1^i^	142.76 (6)	N1—C34—C35	124.1 (4)
O1—Eu1—Eu1^i^	144.67 (6)	N1—C34—H34A	118.0
O3—Eu1—Eu1^i^	80.04 (5)	C35—C34—H34A	118.0
N1—Eu1—Eu1^i^	116.10 (5)	C36—C35—C34	117.9 (4)
N2—Eu1—Eu1^i^	111.84 (5)	C36—C35—H35A	121.0
C1—Eu1—Eu1^i^	153.62 (6)	C34—C35—H35A	121.0
O4^i^—Eu1—Eu1^i^	33.34 (4)	C35—C36—C37	120.9 (4)
C12—Eu1—Eu1^i^	57.10 (6)	C35—C36—H36A	119.6
O1—C1—O2	120.0 (3)	C37—C36—H36A	119.6
O1—C1—C2	119.5 (3)	C36—C37—C45	117.5 (4)
O2—C1—C2	120.5 (3)	C36—C37—C38	124.2 (4)
O1—C1—Eu1	61.21 (15)	C45—C37—C38	118.3 (4)
O2—C1—Eu1	58.82 (15)	C39—C38—C37	122.0 (4)
C2—C1—Eu1	177.1 (2)	C39—C38—H38A	119.0
C3—C2—C11	119.7 (3)	C37—C38—H38A	119.0
C3—C2—C1	117.2 (3)	C38—C39—C40	121.0 (4)
C11—C2—C1	123.1 (3)	C38—C39—H39A	119.5
C2—C3—C4	121.0 (3)	C40—C39—H39A	119.5
C2—C3—H3A	119.5	C41—C40—C44	117.5 (4)
C4—C3—H3A	119.5	C41—C40—C39	122.8 (4)
C5—C4—C3	120.2 (3)	C44—C40—C39	119.6 (4)
C5—C4—H4A	119.9	C42—C41—C40	119.3 (4)
C3—C4—H4A	119.9	C42—C41—H41A	120.3
C4—C5—C6	122.2 (3)	C40—C41—H41A	120.3
C4—C5—H5A	118.9	C41—C42—C43	119.9 (4)
C6—C5—H5A	118.9	C41—C42—H42A	120.1
C7—C6—C5	121.9 (3)	C43—C42—H42A	120.1
C7—C6—C11	120.0 (3)	N2—C43—C42	122.9 (4)
C5—C6—C11	118.1 (3)	N2—C43—H43A	118.6
C8—C7—C6	120.7 (3)	C42—C43—H43A	118.6
C8—C7—H7A	119.6	N2—C44—C40	122.4 (3)
C6—C7—H7A	119.6	N2—C44—C45	118.6 (3)
C7—C8—O9	124.7 (3)	C40—C44—C45	119.0 (3)
C7—C8—C9	119.9 (3)	N1—C45—C37	121.7 (3)
O9—C8—C9	115.4 (3)	N1—C45—C44	118.4 (3)
C10—C9—C8	120.9 (3)	C37—C45—C44	120.0 (3)
C10—C9—H9A	119.5	C34—N1—C45	118.0 (3)
C8—C9—H9A	119.5	C34—N1—Eu1	122.3 (2)
C9—C10—C11	121.9 (3)	C45—N1—Eu1	119.7 (2)
C9—C10—H10A	119.1	C43—N2—C44	117.9 (3)
C11—C10—H10A	119.1	C43—N2—Eu1	122.5 (2)
C10—C11—C2	124.8 (3)	C44—N2—Eu1	119.4 (2)
C10—C11—C6	116.6 (3)	C1—O1—Eu1	92.23 (18)
C2—C11—C6	118.6 (3)	C1—O2—Eu1	94.56 (18)
O4^i^—C12—O3	120.2 (3)	C12—O3—Eu1	106.07 (18)
O4^i^—C12—C13	120.9 (3)	C12^i^—O4—Eu1	167.9 (2)
O3—C12—C13	118.7 (3)	C12^i^—O4—Eu1^i^	84.49 (18)
O4^i^—C12—Eu1	71.56 (17)	Eu1—O4—Eu1^i^	103.52 (7)
O3—C12—Eu1	50.68 (15)	C23—O5—Eu1^i^	144.72 (19)
C13—C12—Eu1	159.4 (2)	C23—O6—Eu1	135.27 (19)
C22—C13—C14	121.0 (3)	C17—O7—H7	109.5
C22—C13—C12	117.9 (3)	C30—O8—H8	109.5
C14—C13—C12	121.0 (3)	C8—O9—H9	109.5
C13—C14—C19	118.6 (3)	H1W—O1W—H2W	113.7
C13—C14—C15	123.5 (3)		
			
O5^i^—Eu1—C1—O1	162.80 (17)	C39—C40—C41—C42	−178.1 (4)
O4—Eu1—C1—O1	−95.5 (3)	C40—C41—C42—C43	0.1 (6)
O6—Eu1—C1—O1	16.2 (2)	C41—C42—C43—N2	−1.5 (6)
O2—Eu1—C1—O1	−177.2 (3)	C41—C40—C44—N2	−0.5 (5)
O3—Eu1—C1—O1	89.65 (19)	C39—C40—C44—N2	178.4 (3)
N1—Eu1—C1—O1	−58.69 (18)	C41—C40—C44—C45	−179.1 (3)
N2—Eu1—C1—O1	−122.27 (18)	C39—C40—C44—C45	−0.2 (5)
O4^i^—Eu1—C1—O1	91.59 (19)	C36—C37—C45—N1	−1.0 (5)
C12—Eu1—C1—O1	86.97 (19)	C38—C37—C45—N1	179.0 (3)
Eu1^i^—Eu1—C1—O1	94.2 (2)	C36—C37—C45—C44	178.9 (3)
O5^i^—Eu1—C1—O2	−20.0 (2)	C38—C37—C45—C44	−1.1 (5)
O4—Eu1—C1—O2	81.7 (3)	N2—C44—C45—N1	2.3 (4)
O6—Eu1—C1—O2	−166.67 (17)	C40—C44—C45—N1	−179.0 (3)
O1—Eu1—C1—O2	177.2 (3)	N2—C44—C45—C37	−177.6 (3)
O3—Eu1—C1—O2	−93.20 (19)	C40—C44—C45—C37	1.1 (4)
N1—Eu1—C1—O2	118.46 (18)	C35—C34—N1—C45	−0.2 (5)
N2—Eu1—C1—O2	54.88 (18)	C35—C34—N1—Eu1	178.0 (3)
O4^i^—Eu1—C1—O2	−91.26 (19)	C37—C45—N1—C34	1.1 (4)
C12—Eu1—C1—O2	−95.88 (18)	C44—C45—N1—C34	−178.8 (3)
Eu1^i^—Eu1—C1—O2	−88.6 (2)	C37—C45—N1—Eu1	−177.2 (2)
O1—C1—C2—C3	23.8 (4)	C44—C45—N1—Eu1	3.0 (3)
O2—C1—C2—C3	−156.6 (3)	O5^i^—Eu1—N1—C34	−157.7 (2)
O1—C1—C2—C11	−156.7 (3)	O4—Eu1—N1—C34	−103.0 (2)
O2—C1—C2—C11	22.8 (5)	O6—Eu1—N1—C34	−23.9 (2)
C11—C2—C3—C4	−0.9 (5)	O2—Eu1—N1—C34	110.8 (2)
C1—C2—C3—C4	178.6 (3)	O1—Eu1—N1—C34	63.8 (2)
C2—C3—C4—C5	2.6 (6)	O3—Eu1—N1—C34	29.8 (3)
C3—C4—C5—C6	−1.5 (7)	N2—Eu1—N1—C34	177.6 (3)
C4—C5—C6—C7	178.2 (4)	C1—Eu1—N1—C34	87.2 (2)
C4—C5—C6—C11	−1.3 (6)	O4^i^—Eu1—N1—C34	−50.3 (3)
C5—C6—C7—C8	179.5 (3)	C12—Eu1—N1—C34	−10.6 (3)
C11—C6—C7—C8	−1.0 (5)	Eu1^i^—Eu1—N1—C34	−79.8 (2)
C6—C7—C8—O9	−179.7 (3)	O5^i^—Eu1—N1—C45	20.5 (3)
C6—C7—C8—C9	−0.5 (5)	O4—Eu1—N1—C45	75.2 (2)
C7—C8—C9—C10	1.7 (5)	O6—Eu1—N1—C45	154.2 (2)
O9—C8—C9—C10	−179.0 (3)	O2—Eu1—N1—C45	−71.0 (2)
C8—C9—C10—C11	−1.3 (5)	O1—Eu1—N1—C45	−118.0 (2)
C9—C10—C11—C2	177.6 (3)	O3—Eu1—N1—C45	−152.02 (19)
C9—C10—C11—C6	−0.2 (5)	N2—Eu1—N1—C45	−4.2 (2)
C3—C2—C11—C10	−179.6 (3)	C1—Eu1—N1—C45	−94.6 (2)
C1—C2—C11—C10	0.9 (5)	O4^i^—Eu1—N1—C45	127.9 (2)
C3—C2—C11—C6	−1.8 (5)	C12—Eu1—N1—C45	167.61 (19)
C1—C2—C11—C6	178.7 (3)	Eu1^i^—Eu1—N1—C45	98.4 (2)
C7—C6—C11—C10	1.3 (4)	C42—C43—N2—C44	1.8 (5)
C5—C6—C11—C10	−179.2 (3)	C42—C43—N2—Eu1	−173.8 (3)
C7—C6—C11—C2	−176.6 (3)	C40—C44—N2—C43	−0.8 (4)
C5—C6—C11—C2	2.9 (5)	C45—C44—N2—C43	177.9 (3)
O5^i^—Eu1—C12—O4^i^	62.03 (17)	C40—C44—N2—Eu1	174.9 (2)
O4—Eu1—C12—O4^i^	−9.1 (2)	C45—C44—N2—Eu1	−6.4 (3)
O6—Eu1—C12—O4^i^	−81.16 (18)	O5^i^—Eu1—N2—C43	18.3 (2)
O2—Eu1—C12—O4^i^	143.34 (17)	O4—Eu1—N2—C43	96.0 (2)
O1—Eu1—C12—O4^i^	−163.24 (17)	O6—Eu1—N2—C43	150.5 (2)
O3—Eu1—C12—O4^i^	163.6 (3)	O2—Eu1—N2—C43	−69.4 (2)
N1—Eu1—C12—O4^i^	−95.0 (2)	O1—Eu1—N2—C43	−114.6 (2)
N2—Eu1—C12—O4^i^	70.1 (2)	O3—Eu1—N2—C43	−31.9 (3)
C1—Eu1—C12—O4^i^	170.14 (18)	N1—Eu1—N2—C43	−179.0 (3)
Eu1^i^—Eu1—C12—O4^i^	−6.04 (14)	C1—Eu1—N2—C43	−91.8 (2)
O5^i^—Eu1—C12—O3	−101.6 (2)	O4^i^—Eu1—N2—C43	43.9 (3)
O4—Eu1—C12—O3	−172.7 (2)	C12—Eu1—N2—C43	10.3 (3)
O6—Eu1—C12—O3	115.2 (2)	Eu1^i^—Eu1—N2—C43	71.7 (2)
O2—Eu1—C12—O3	−20.3 (2)	O5^i^—Eu1—N2—C44	−157.2 (2)
O1—Eu1—C12—O3	33.1 (2)	O4—Eu1—N2—C44	−79.5 (2)
N1—Eu1—C12—O3	101.3 (2)	O6—Eu1—N2—C44	−25.0 (2)
N2—Eu1—C12—O3	−93.5 (3)	O2—Eu1—N2—C44	115.1 (2)
C1—Eu1—C12—O3	6.5 (2)	O1—Eu1—N2—C44	69.9 (2)
O4^i^—Eu1—C12—O3	−163.6 (3)	O3—Eu1—N2—C44	152.55 (19)
Eu1^i^—Eu1—C12—O3	−169.7 (2)	N1—Eu1—N2—C44	5.44 (19)
O5^i^—Eu1—C12—C13	−167.1 (6)	C1—Eu1—N2—C44	92.7 (2)
O4—Eu1—C12—C13	121.7 (6)	O4^i^—Eu1—N2—C44	−131.59 (19)
O6—Eu1—C12—C13	49.7 (6)	C12—Eu1—N2—C44	−165.26 (19)
O2—Eu1—C12—C13	−85.8 (6)	Eu1^i^—Eu1—N2—C44	−103.8 (2)
O1—Eu1—C12—C13	−32.4 (6)	O2—C1—O1—Eu1	−2.8 (3)
O3—Eu1—C12—C13	−65.5 (6)	C2—C1—O1—Eu1	176.7 (2)
N1—Eu1—C12—C13	35.8 (6)	O5^i^—Eu1—O1—C1	−22.9 (2)
N2—Eu1—C12—C13	−159.0 (5)	O4—Eu1—O1—C1	144.29 (18)
C1—Eu1—C12—C13	−59.0 (6)	O6—Eu1—O1—C1	−164.72 (19)
O4^i^—Eu1—C12—C13	130.8 (7)	O2—Eu1—O1—C1	1.60 (17)
Eu1^i^—Eu1—C12—C13	124.8 (6)	O3—Eu1—O1—C1	−82.84 (18)
O4^i^—C12—C13—C22	95.3 (4)	N1—Eu1—O1—C1	117.37 (19)
O3—C12—C13—C22	−81.4 (4)	N2—Eu1—O1—C1	60.16 (19)
Eu1—C12—C13—C22	−28.0 (7)	O4^i^—Eu1—O1—C1	−103.32 (18)
O4^i^—C12—C13—C14	−86.8 (4)	C12—Eu1—O1—C1	−95.73 (18)
O3—C12—C13—C14	96.6 (4)	Eu1^i^—Eu1—O1—C1	−129.97 (16)
Eu1—C12—C13—C14	150.0 (5)	O1—C1—O2—Eu1	2.9 (3)
C22—C13—C14—C19	0.4 (5)	C2—C1—O2—Eu1	−176.7 (2)
C12—C13—C14—C19	−177.5 (3)	O5^i^—Eu1—O2—C1	161.01 (19)
C22—C13—C14—C15	−179.8 (3)	O4—Eu1—O2—C1	−148.32 (17)
C12—C13—C14—C15	2.3 (5)	O6—Eu1—O2—C1	18.0 (2)
C13—C14—C15—C16	−178.8 (3)	O1—Eu1—O2—C1	−1.59 (17)
C19—C14—C15—C16	1.0 (5)	O3—Eu1—O2—C1	79.71 (18)
C14—C15—C16—C17	−0.5 (5)	N1—Eu1—O2—C1	−62.98 (19)
C15—C16—C17—C18	−0.4 (6)	N2—Eu1—O2—C1	−121.79 (19)
C15—C16—C17—O7	179.8 (3)	O4^i^—Eu1—O2—C1	103.63 (18)
O7—C17—C18—C19	−179.6 (3)	C12—Eu1—O2—C1	87.79 (18)
C16—C17—C18—C19	0.7 (6)	Eu1^i^—Eu1—O2—C1	132.77 (16)
C17—C18—C19—C20	−179.7 (3)	O4^i^—C12—O3—Eu1	−18.0 (4)
C17—C18—C19—C14	−0.1 (5)	C13—C12—O3—Eu1	158.6 (2)
C13—C14—C19—C18	179.1 (3)	O5^i^—Eu1—O3—C12	73.3 (2)
C15—C14—C19—C18	−0.7 (5)	O4—Eu1—O3—C12	8.5 (2)
C13—C14—C19—C20	−1.4 (5)	O6—Eu1—O3—C12	−59.3 (2)
C15—C14—C19—C20	178.8 (3)	O2—Eu1—O3—C12	159.4 (2)
C18—C19—C20—C21	−179.5 (4)	O1—Eu1—O3—C12	−145.7 (2)
C14—C19—C20—C21	1.0 (5)	N1—Eu1—O3—C12	−112.0 (2)
C19—C20—C21—C22	0.4 (6)	N2—Eu1—O3—C12	122.5 (2)
C14—C13—C22—C21	0.9 (5)	C1—Eu1—O3—C12	−173.3 (2)
C12—C13—C22—C21	178.9 (3)	O4^i^—Eu1—O3—C12	9.05 (19)
C20—C21—C22—C13	−1.4 (6)	Eu1^i^—Eu1—O3—C12	8.8 (2)
O5—C23—C24—C25	−145.8 (3)	O5^i^—Eu1—O4—C12^i^	63.5 (10)
O6—C23—C24—C25	31.7 (5)	O6—Eu1—O4—C12^i^	−159.5 (10)
O5—C23—C24—C33	29.7 (4)	O2—Eu1—O4—C12^i^	10.7 (11)
O6—C23—C24—C33	−152.9 (3)	O1—Eu1—O4—C12^i^	−107.0 (10)
C33—C24—C25—C26	−2.8 (7)	O3—Eu1—O4—C12^i^	130.8 (10)
C23—C24—C25—C26	172.7 (4)	N1—Eu1—O4—C12^i^	−80.8 (10)
C24—C25—C26—C27	0.7 (8)	N2—Eu1—O4—C12^i^	−15.6 (10)
C25—C26—C27—C28	0.9 (9)	C1—Eu1—O4—C12^i^	−43.3 (12)
C26—C27—C28—C29	179.5 (5)	O4^i^—Eu1—O4—C12^i^	130.4 (11)
C26—C27—C28—C33	−0.3 (8)	C12—Eu1—O4—C12^i^	134.2 (10)
C27—C28—C29—C30	−176.8 (4)	Eu1^i^—Eu1—O4—C12^i^	130.4 (11)
C33—C28—C29—C30	3.0 (6)	O5^i^—Eu1—O4—Eu1^i^	−66.92 (8)
C28—C29—C30—O8	−179.0 (3)	O6—Eu1—O4—Eu1^i^	70.03 (7)
C28—C29—C30—C31	0.3 (6)	O2—Eu1—O4—Eu1^i^	−119.73 (13)
C29—C30—C31—C32	−2.5 (5)	O1—Eu1—O4—Eu1^i^	122.51 (14)
O8—C30—C31—C32	176.8 (3)	O3—Eu1—O4—Eu1^i^	0.38 (12)
C30—C31—C32—C33	1.5 (5)	N1—Eu1—O4—Eu1^i^	148.79 (9)
C31—C32—C33—C28	1.8 (5)	N2—Eu1—O4—Eu1^i^	−146.04 (9)
C31—C32—C33—C24	179.2 (3)	C1—Eu1—O4—Eu1^i^	−173.7 (3)
C27—C28—C33—C32	175.8 (4)	O4^i^—Eu1—O4—Eu1^i^	0.0
C29—C28—C33—C32	−3.9 (5)	C12—Eu1—O4—Eu1^i^	3.80 (9)
C27—C28—C33—C24	−1.8 (6)	O6—C23—O5—Eu1^i^	−9.4 (6)
C29—C28—C33—C24	178.4 (3)	C24—C23—O5—Eu1^i^	167.9 (2)
C25—C24—C33—C32	−174.1 (4)	O5—C23—O6—Eu1	−21.9 (5)
C23—C24—C33—C32	10.6 (5)	C24—C23—O6—Eu1	160.9 (2)
C25—C24—C33—C28	3.3 (5)	O5^i^—Eu1—O6—C23	36.2 (3)
C23—C24—C33—C28	−172.0 (3)	O4—Eu1—O6—C23	−21.9 (3)
N1—C34—C35—C36	−0.8 (6)	O2—Eu1—O6—C23	165.7 (3)
C34—C35—C36—C37	0.8 (6)	O1—Eu1—O6—C23	−178.6 (3)
C35—C36—C37—C45	0.0 (5)	O3—Eu1—O6—C23	105.4 (3)
C35—C36—C37—C38	180.0 (4)	N1—Eu1—O6—C23	−103.8 (3)
C36—C37—C38—C39	−179.8 (4)	N2—Eu1—O6—C23	−76.0 (3)
C45—C37—C38—C39	0.1 (6)	C1—Eu1—O6—C23	174.2 (3)
C37—C38—C39—C40	0.8 (7)	O4^i^—Eu1—O6—C23	58.5 (3)
C38—C39—C40—C41	178.1 (4)	C12—Eu1—O6—C23	84.2 (3)
C38—C39—C40—C44	−0.7 (6)	Eu1^i^—Eu1—O6—C23	22.4 (3)
C44—C40—C41—C42	0.8 (5)		

Symmetry codes: (i) −*x*+1, −*y*+1, −*z*+1.

### Hydrogen-bond geometry (Å, °)

**Table:**

*D*—H···*A*	*D*—H	H···*A*	*D*···*A*	*D*—H···*A*
O1W—H1W···O9^ii^	0.85	2.05	2.799 (4)	147
O1W—H2W···O8^iii^	0.85	2.28	3.050 (4)	150
O7—H7···O1W	0.82	1.89	2.670 (5)	159
O8—H8···O7^iv^	0.82	1.88	2.664 (5)	160
O9—H9···O3^v^	0.82	1.84	2.642 (3)	165

Symmetry codes: (ii) *x*, *y*+1, *z*; (iii) −*x*+1, −*y*+2, −*z*+1; (iv) *x*−1, *y*+1, *z*−1; (v) −*x*+1, −*y*, −*z*+2.
